# Bacterial Aetiologies of Lower Respiratory Tract Infections among Adults in Yaoundé, Cameroon

**DOI:** 10.1155/2019/4834396

**Published:** 2019-04-17

**Authors:** Serges Tchatchouang, Ariane Nzouankeu, Sebastien Kenmoe, Laure Ngando, Veronique Penlap, Marie-Christine Fonkoua, Eric-Walter Pefura-Yone, Richard Njouom

**Affiliations:** ^1^Department of Virology, Centre Pasteur of Cameroon, Member of the International Network of Pasteur Institutes, P.O. Box 1274, Yaoundé, Cameroon; ^2^Department of Bacteriology, Centre Pasteur of Cameroon, Member of the International Network of Pasteur Institutes, P.O. Box 1274, Yaoundé, Cameroon; ^3^Department of Biochemistry, Faculty of Science, University of Yaoundé 1, P.O. Box 812, Yaoundé, Cameroon; ^4^Department of Pneumology, Jamot Hospital, P.O. Box 4021, Yaoundé, Cameroon

## Abstract

Lower respiratory tract infections (LRTIs) remain a challenge in African healthcare settings and only few data are available on their aetiology in Cameroon. The purpose of this study was to access the bacterial cause of LRTIs in patients in Cameroon by two methods.* Methods*. Participants with LRTIs were enrolled in the referral centre for respiratory diseases in Yaoundé city and its surroundings. To detect bacteria, specimens were tested by conventional bacterial culture and a commercial reverse-transcriptase real-time polymerase chain reaction (RT-PCR) assay. One hundred forty-one adult patients with LRTIs were enrolled in the study. Among the participants, 46.8% were positive for at least one bacterium.* Streptococcus pneumoniae* and* Haemophilus influenzae *were the most detected bacteria with 14.2% (20/141) followed by* Klebsiella pneumoniae*, 9.2% (13/141),* Staphylococcus aureus*, 7.1% (10/141), and* Moraxella catarrhalis*, 4.3% (6/141). Bacterial coinfection accounted for 23% (14/61) with* Haemophilus influenzae *being implicated in 19.7% (12/61). The diagnostic performance of RT-PCR for bacteria detection (43.3%) was significantly different from that of culture (17.7%) (p< 0.001). Only* Streptococcus pneumoniae *detection was associated with empyema by RT-PCR (p<0.001). These findings enhance understanding of bacterial aetiologies in order to improve respiratory infection management and treatment. It also highlights the need to implement molecular tools as part of the diagnosis of LRTIs.

## 1. Introduction

Lower respiratory tract infections (LRTIs) are major cause of morbidity and mortality globally [1]. In Africa, they are one of the most prevalent causes of death [2]. Particularly in sub-Saharan Africa, high case fatality ratios were reported in Somalia and Chad with 546.8 and 511.3 deaths per 100 000 inhabitants, respectively, when compared to the lowest mortality (0.65 deaths per 100 000 inhabitants) registered in Finland (Europe) [2]. LRTI is a broad terminology encompassing different clinical presentations and aetiologies, which may vary according to, for example, age and season among others [3]. Overall, viruses are responsible for a large proportion of LRTIs but antibiotics are often unnecessarily prescribed for their treatment without any laboratory testing [4, 5] and can contribute to the emergence of antimicrobial resistance [6]. Other causes of LRTIs are bacteria:* Streptococcus pneumoniae, Haemophilus influenzae, Klebsiella pneumoniae*, and* Staphylococcus aureus *being the most common [2, 7, 8].

However, studies on bacterial aetiologies of LRTIs in Cameroon are limited. In addition, the few studies performed in the 90's used traditional culture methods [9, 10]. Although culture is still considered to be the gold standard, the method has important disadvantages, such as a longer time to result, the stringent specimen collection and transport condition and the risk of inhibited growth of the pathogens due to previous antibiotic treatment [11, 12]. As a consequence, many patients in African healthcare centres remain undiagnosed despite clinical evidence of LRTIs.

The development of nucleic acid amplification tests (NAATs) has revolutionized clinical bacteriology [13]. They are promising alternative diagnostic methods. Compared to culture, they are able to provide results within a few hours, are sensitive, and do not require viable organisms [14, 15]. Using NAATs, the viral aetiology of respiratory infections in Cameroon has been largely documented [16, 17]; meanwhile bacterial aetiology on the other hand remains unexplored. Knowing the aetiology of LRTIs can help avoid unnecessary antibiotherapy in healthcare settings.

Our overall aim was to identify the respiratory bacteria of patients presenting with symptoms and clinical signs of LRTI at a referral centre for respiratory diseases in Yaoundé, Cameroon. Here we report on the bacteria that were detected using a commercial real-time polymerase chain reaction (RT-PCR) assay in addition to traditional culture methods.

## 2. Methodology

### 2.1. Study Design

We conducted a prospective study among patients presenting with symptoms and clinical signs of LRTI at the pneumology department of the Jamot Hospital in Yaoundé from mid-January 2017 to mid-January 2018. This hospital is the management centre for respiratory diseases of Yaoundé city and its surroundings. LTRIs were cases of bronchitis, bronchiolitis, and pneumonia diagnosed by the physician. Adult patients who presented at least two of the following symptoms were included in the study after written informed consent was obtained: fever, cough, dyspnoea, wheezing, chest pain, or sore throat. Any prior antimicrobial treatment taken by the patient was also recorded before microbiological investigations. Patients diagnosed with pulmonary tuberculosis or with infections other than LRTIs were excluded. Sociodemographic data and clinical signs were recorded before enrolment. Empiric antibiotic therapy was not administered to patients before laboratory investigation. To guide antibiotic selection in the follow-up of participants, drug susceptibility testing was done and provided to the physicians but is not presented here.

### 2.2. Ethics and Consent to Participate

The study was reviewed and approved by the participating hospital and the National Research Ethics Committee of Cameroon (N°2017/03/876/CE/CNERSH/SP). All participants and/or the parents/legal guardians of minors provided written informed consent before enrolment. In addition, assent was sought for participants below the age of 21 year, legal age of majority in Cameroon.

### 2.3. Sample Collection

Clinical samples consisted of bronchoalveolar lavages (BALs) and fluids drained from pleural effusion (FPEf) or pleural empyema (FPEm) depending on the clinical and radiological presentation of the patients. Empyema was considered as collection of pus in the pleural space with secondary inflammation of the visceral and parietal pleura [18]. The samples were transported at ambient temperature and within 1 hour after collection to Centre Pasteur of Cameroon (CPC) for diagnostic testing (microscopy, bacterial culture, and anatomopathology analysis for FPEf). Upon receipt at the CPC, the samples were divided into two aliquots. One aliquot was immediately used for bacterial culture; the other was stored in medium (universal transport medium) and kept at -80°C until molecular amplification.

### 2.4. Bacterial Culture

According to the French REMIC guidelines, a loopful (10 *μ*L) of the sample (BAL and pleural fluids) was plated onto chocolate, 5% sheep blood agar (BA) media, and Cysteine-Lactose-Electrolyte-Deficient (CLED) plates. After inoculation, the agar plates were incubated at 37°C, with the chocolate and BA agar plates in a 5% CO_2_ atmosphere and the CLED agar in normal atmosphere, for 18 to 24 hours. For isolation of anaerobic bacteria, FPEm was inoculated onto 5% sheep blood agar plates and was incubated at 37°C for 18 to 72 hours using GENbag anaer (bioMérieux, Marcy l'Etoile, France) to generate anaerobic growth conditions.

For pleural fluids, all growth bacteria were considered as positive irrespective of number of colonies. For BAL, protected bronchoalveolar lavage was done to avoid contamination by the oropharyngeal commensal flora. BAL fluids were serially diluted (dilutions of 1:10, 1:100, and 1:1000) and bacterial growth was defined as significant when present as ≥ 10^4^ colony forming units (cfu) per mL.

Isolated colonies were identified using Gram staining, common biochemical tests [19], and the Vitek Compact 2 system (bioMérieux, Marcy l'Etoile, France).

### 2.5. RNA Extraction

RNA was extracted from all samples using the QIAamp Viral RNA Mini kit (Qiagen, Hilden, Germany), following the manufacturer's instructions. A final elution volume of 60 *μ*L of RNA was stored in 1.5 mL Eppendorf tubes at –20°C until amplification.

### 2.6. Molecular Amplification

The RNAs were tested using the commercial RT-PCR assay Fast-track Diagnostics Respiratory pathogens (Fast-track Diagnostics, Junglinster, Luxembourg). This kit enabled targeting 10 bacteria:* Mycoplasma pneumoniae, Chlamydophila pneumoniae, Streptococcus pneumoniae, Haemophilus influenzae type b, Staphylococcus aureus, Klebsiella pneumoniae, Legionella pneumophila/longbeachae, Salmonella *spp.*, Moraxella catarrhalis, Bordetella *spp. (except* Bordetella parapertussis*), and* Haemophilus influenzae. *The manufacturer's instructions were followed, briefly: a volume of 10 *μ*L of RNA was added to 15 *μ*L of fast tract master mix (buffer, primers, probes, and enzyme). Amplification was performed in an ABI PRISM 7500 RT-PCR machine (Applied Biosystems, Foster City, CA, USA). An internal control was added to all samples to ensure validity of the assay. Positive and negative controls were included in each experiment.

### 2.7. Statistical Analysis

Data were analysed using the Statistical Package for Social Sciences software (version 22.0, SPSS Inc., Chicago, IL, USA). Distribution of categorical variables was compared using the chi-square or Fisher's exact tests as appropriate. Independent* t*-test was done for mean comparison between groups. Results of bacterial detection by culture and RT-PCR were compared using the McNemar test. The significance level was set at 0.05.

## 3. Results

### 3.1. Study Population

Overall, a total of 141 patients were enrolled; the male/female sex ratio was 1.8. The patients' age ranged from 18 to 94 years with a median age of 50 years (interquartile range: 34.7-62.1). No mean age difference was noticed among males and females (p= 0.786). The sociodemographic and clinical characteristics of the patients are summarised in Table 1. The most predominant symptoms were cough (87.2%), dyspnoea (85.8%), breathlessness (83%), asthenia (75.9%), fever (63.8%), chest pain (60.3%), and myalgia (42.6%). Among the type of specimens collected, FPEf accounted for 47.1% (67/141), BAL for 28.4% (40/141), and FPEm for 24.1% (34/141). The majority of patients (76.6%) had already taken antimicrobials (beta-lactams, sulfonamides, aminoglycoside, macrolides, and quinolones) before the enrolment.

### 3.2. Bacterial Detection

Bacteria were detected in 17.7% (25/141) and 43.3% (61/141) of the samples using traditional culture and RT-PCR, respectively (p<0.001). Combining both techniques, an aetiological bacterial agent was detected in 46.8% (66/141) of the samples. Both methods detected more frequently* S. pneumoniae *(14.2% by RT-PCR versus 5.7% by culture),* H. influenzae *(14.2% by RT-PCR versus 3.5% by culture), and* K. pneumoniae *(9.2% by RT-PCR versus 3.5% by culture).

As the BALs were sampled aseptically, we obtained 14 bacterial growths out of the 40 plated. One culture exhibited two bacterial species out of 14. The BAL cultures for which the less bacterial growth was noted had 10^4^ cfu/mL. For pleural fluids, the bacterial growth ranged from 7 to more than 10^6^ cfu/mL.

There was no association between clinical symptoms of inclusion and bacterial detection methods (p>0.05). There was an association between myalgia and bacterial culture (p= 0.014). There was no difference for bacterial detection among patients under antibiotics prior to the diagnosis and those who were not (p=0.757). Results according to sample type and method are presented in Table 2.* S. pneumoniae *was significantly more detected in FPEm (p<0.001) compared to the other sample types.

### 3.3. Coinfections

Multiple bacteria or bacterial coinfections were detected in a total of 14/61 (23%) specimens using RT-PCR. Only one case of coinfection was reported with culture:* Pseudomonas aeruginosa* and* Citrobacter koseri *(4%; 1/25) in BAL.  Table 3 shows the different coinfections obtained according to the clinical sample.

## 4. Discussion

The diagnosis and management of respiratory tract infections are great challenges in Africa due to the socioeconomic burden and limited access to good healthcare and hospitals. In order to formulate adequate guideline for the management of LRTIs, including diagnosis and treatment, data on the aetiology of the LRTIs should be obtained. This study focused on establishing the bacterial aetiology of LRTIs following diagnostic tools and type of clinical samples. One hundred and forty-one clinical samples from patients presenting symptoms of LRTIs were subjected to bacterial investigations using traditional culture and RT-PCR.

At least one bacterium could be detected in 46.8% of the 141 patients clinically diagnosed with LRTIs. This prevalence is similar to 45.2% found in Enugu State, Nigeria [20], but higher than the 24% reported in Tunisia [21]. Using the same diagnostic approach, prevalence of 77% and 85.7% was reported in Gambia and in Osun State, Nigeria, respectively [22, 23]. The prevalence rates may be explained by the differences in study designs and geographic areas. Indeed, the spread of respiratory infections varies between populations and countries, depending on difference in geography, climate, and socioeconomic conditions [24–27]. In addition, we reported the bacterial aetiology of LRTIs in adults, whereas most studies included only children. The lower prevalence rate compared to Gambia and Nigeria might also be explained by the high percentage (76.6%) of patients who used antibiotics before enrolment.

*S. pneumoniae* was the leading pathogen of LRTIs followed by* H. influenzae *and* K. pneumoniae. S. pneumoniae *was also found to be the main cause of LRTIs in a study in Malawi [28] and Tunisia [21]. However, other studies in Nigeria, which is a neighboring country of Cameroon, reported* K. pneumoniae *as the most detected pathogen [8, 20] in LRTIs or* S. aureus* in Tunisia [29]*. S. pneumoniae *remains an important pathogen in LRTIs even with the introduction of 13-valent pneumococcal conjugate vaccine [30]. This suggests that further investigations are needed for vaccine impact. Conversely, no* H. influenzae* type b was observed. Although* S. pneumoniae, H. influenzae*, and* K. pneumoniae* were the key bacteria, there was a remarkable variation in distribution of these etiologic agents between clinical samples.

We observed an association between bacterial detection and type of clinical sample. The bacterial detection rate was the lowest in FPEf compared to the other clinical samples. The frequency of bacterial detection in FPEf (3% by culture) was as low as the one observed in Spain, where 7% (14/191) of fluids from uncomplicated parapneumonic pleural effusions were culture-positive [31]. The low detection level could be due to the fact that 80.6% of FPEf were from patients undergoing an antimicrobial treatment.

In FPEm, the bacterial detection rate by culture (26.5%) was low compared to previous results obtained in the Jamot Hospital (Yaoundé, Cameroon) 8 years ago, where the bacterial detection rate was 53.7% [32] and also compared to a study in Lleida Spain, reporting a culture positivity rate of 66% [31]. But our bacterial detection rate in FPEm was higher compared to the one obtained in San Sebastian, Spain, where only 10% of bacteria-positive FPEm was detected by culture between January 2005 and December 2012 [33]. Also in the study performed in San Sebastian, the use of antibiotics prior to bacteriological investigations was high (81.7%). However, the study performed in Lleida did not report on the prior use of antibiotics. Most bacterial detections were in FPEm and we hypothesize that this could be due to the nutrient-rich medium of FPEm supporting the growth of pathogenic bacteria such as* S*.* pneumoniae* strains. This may be one of the reasons why pneumococci are a common bacterial cause of empyema. Thus, the high density and persistent growth of* S. pneumoniae *in pleural fluid highlight the importance of draining infected effusions [34].

We found* H. influenzae* to be the key pathogen in BAL, which is different from what has been observed in lung aspirates of Malawian and Gambian children with* S. pneumoniae *[22, 35]. Our result in BAL is also different from the ones in Korean adults with methicillin-resistant* S. aureus *[36] as the most frequently isolated bacterium. Overall, bacterial profiles from BAL vary among studies [37].

The RT-PCR increased the yield of bacteria detection by 25.5%. As previously reported [33], the use of molecular tool has greatly improved microbial diagnosis of LRTIs. In the present study, only 25 positive cultures were obtained among the 141 patients. Concordant with a recent study conducted in Sweden [38], using the RT-PCR, we were able to detect the noncultivable and fastidious pathogen* Legionella species* which is overlooked by routine standard culture. Rapid detection of uncommon pathogens from LRTI samples by molecular tool might be important in the clinical routine. Almost all coinfections were detected using RT-PCR. Bacterial coinfection rate was near to the 18.9% of inpatients with nonresponding community-acquired pneumonia in Belgium [39]. This finding suggests that molecular assay is the most useful method to detect coinfections representing near-past and current infections.

*Limitations. *Antibiotic treatment prior to the diagnosis and delay of patients could be biases for real pathogen frequencies reported in this study.

## 5. Conclusion

Bacterial aetiology was more reported in cases of LRTI with empyema.* S. pneumoniae* and* H. influenzae* were the most commonly detected bacteria. The molecular tool used during investigation detected significantly more bacteria than traditional culture and may be an additional helpful tool for diagnosis of LRTIs. Nevertheless, control of antibiotic use and early diagnosis are crucial when managing LRTIs.

## Figures and Tables

**Table 1:** Sociodemographic and clinical characteristics of the study population.

Study population	Total (n=141)	Positive bacterial culture (n=25)
Age		
Median age in years (IQR)	50 (34.7- 62.1)	48 (32.9-62.8)
18-35 years (young adults)	37 (26.2)	8 (32)
36-55 years (middle-aged adults)	56 (39.7)	9 (36)
>55 years (old adults)	48 (34)	8 (32)
Gender		
Males	90 (63.8)	16 (64)
Females	51 (36.2)	9 (36)
Smoking habit	30 (21.3)	2 (8)
Duration of symptoms (days)	45 (21-90)	45 (37.5-67.7)
Clinical signs		
Cough	123 (87.2)	22 (88)
Dyspnoea	121 (85.8)	22 (88)
Breathlessness	117 (83)	20 (80)
Asthenia	107 (75.9)	19 (76)
Fever	90 (63.8)	16 (64)
Chest pain	85 (60.3)	13 (52)
Myalgia	60 (42.6)	5 (20)
Arthralgia	31 (22)	5 (20)
Headaches	24 (17)	1 (4)
Sore throat	14 (9.9)	2 (8)
Vomiting	11 (7.8)	2 (8)
Diarrhea	10 (7.1)	0
Rhinorrhea	7 (5)	1 (4)
Conjunctivitis	2 (1.4)	0

Data are number and percentage in brackets; IQR: interquartile range; n: number.

**Table 2:** Bacteria detected in fluids from lower respiratory tract by culture and RT-PCR.

	*FPEf (n=67)*		*BAL (n=40)*		*FPEm (n=34)*	
	Culture (%)	RT-PCR (%)	Culture (%)	RT-PCR (%)	Culture (%)	RT-PCR (%)
*Bacteria*						

*Streptococcus pneumoniae*	1 (1.5)	3 (7.1)	2 (5)	4 (10)	5 (14.7)	13 (38.2)
*Haemophilus influenzae*	0	7 (11.4)	4 (10)	9 (22.5)	1 (2.9)	4 (11.7)
*Klebsiella pneumoniae*	1 (1.5)	4 (5.7)	4 (10)	4 (10)	0	5 (14.7)
*Moraxella catarrhalis*	0	0	0	4 (10)	0	2 (5.9)
*Staphylococcus aureus*	0	3 (4.3)	0	3 (7.5)	1 (2.9)	4 (11.7)
*Streptococcus anginosus*	0	/	0	/	1 (2.9)	/
*Streptococcus constellatus*	0	/	0	/	1 (2.9)	/
*Enterobacter agglomerans*	0	/	1 (2.5)	/	0	/
*Pseudomonas aeruginosa*	0	/	2 (5)	/	0	/
*Citrobacter koseri*	0	/	1 (2.5)	/	0	/
*Legionella species*	/	0	/	3 (7.5)	/	3 (8.9)

*Total*	*2 (3)*	*17 (25.4)*	*14 (35)*	*27 (67.5)*	*9 (26.5)*	*31 (91.1)*

BAL: bronchoalveolar lavage; RT-PCR: reverse-transcriptase real-time polymerase chain reaction; FPEf: fluid drained from pleural effusion; FPEm: fluid drained from pleural empyema.

**Table 3:** Level of coinfections in lower respiratory tract by RT-PCR.

Infection level	Pathogens	Number	Clinical samples
Double bacterial infection	*Haemophilus influenzae + Legionella species*	1	FPEm
	*Haemophilus influenzae + Streptococcus pneumoniae*	3	2 BAL + 1 FPEm
	*Streptococcus pneumoniae +Klebsiella pneumoniae*	2	FPEm
	*Haemophilus influenzae + Staphylococcus aureus*	3	FPEf, 2 BAL
	*Haemophilus influenzae +Klebsiella pneumoniae*	2	BAL+ FPEf
Triple bacterial infection	*Haemophilus influenzae + Staphylococcus aureus +Klebsiella pneumoniae*	1	FPEm
	*Haemophilus influenzae + Legionella species + Klebsiella pneumonia*	1	BAL
	*Haemophilus influenzae + Legionella species + Moraxella catarrhalis*	1	FPEm

RT-PCR: reverse-transcriptase real-time polymerase chain reaction; BAL: bronchoalveolar lavage; FPEf: fluid drained from pleural effusion; FPEm: fluid drained from pleural empyema.

## Data Availability

The data used to support the findings of this study are available from the corresponding author upon request.
